# Supplementary material for: Reconstructing Reliable Powder Patterns from Spikelets (Q)CPMG NMR Spectra: Simplification of UWNMR Crystallography Analysis
Source: Molecules. 2021 Oct 6;26(19):6051. doi: 10.3390/molecules26196051 (PMC8513071; doi:10.3390/molecules26196051)
Supplement: Supplementary file 1 [file molecules-26-06051-s001.zip › molecules-1393461_SI_r1.pdf]

Reconstructing Reliable Powder Patterns from Spikelets (Q)CPMG NMR Spectra: Simplification of UWNMR crystallography analysis

Andrii Mahun[1, 2], Sabina Abbrent[1], Jiri Czernek[1], Jan Rohlicek[3], Hana Mackova[1], Weihua Ning[4,†], Rafał Konefał[1], Jiri Brus[1] and Libor Kobera[1]*

[1]Institute of Macromolecular Chemistry of the Czech Academy of Sciences, Heyrovskeho nam. 2, 162 06, Prague 6, Czech Republic

[2]Department of Physical and Macromolecular Chemistry, Faculty of Science, Charles University, Hlavova 8, 128 40, Prague 2, Czech Republic

[3]Department of Structural Analysis, Institute of Physics of the Czech Academy of Sciences, Na Slovance 2, 182 21, Prague 8, Czech Republic

[4]Department of Physics, Chemistry and Biology (IFM), Linköping University, Linköping SE-581 83, Sweden

[†]Current address: Institute of Functional Nano & Soft Materials (FUNSOM), Jiangsu Key Laboratory for Carbon-Based Functional Materials & Devices, Soochow University, Suzhou 215123, PR China

Table of contents

**Table S1.** Details of experimental parameters	S3
**Table S2.** Used external standards for NMR chemical shift referencing	S3
**Table S3.** Crystallographic data of the investigated compounds	S4
**Chart S1.** Description of a procedure for verifying the spectral shape	S5
**Figure S1.** Full experimental ^{137}Ba WURST-QCPMG NMR spectrum of $BaCO_3$ and the spectral profile obtained by the UWNMRSpectralShape software	S5
**Figure S2.** Phase analysis of the measured powder diffraction pattern of $BaCO_3$ sample	S6
**Table S4**. Comparison of NMR parameters obtained by fitting spectral envelopes provided by different approaches for chosen model compounds	S7
**Figure S3.** Experimental ^{209}Bi WURST-QCPMG NMR spectrum of $Cs_2AgBiBr_6$, the spectrum profile obtained by the UWNMRSpectralShape software, and experimental ^{209}Bi Hahn-echo spectrum	S8
**Figure S4.** Phase analysis of the measured powder diffraction of $Cs_2AgBiBr_6$ sample	S8
**Figure S5.** Experimental ^{71}Ga WURST-QCPMG NMR spectrum of $Ga(NO_3)_3$, the spectrum profile obtained by the UWNMRSpectralShape software, and experimental ^{71}Ga Hahn-echo spectrum	S9

**Figure S6.** Phase analysis of the measured powder diffraction pattern of $Ga(NO_3)_3{\cdot}9H_2O$ sample	S9
**Figure S7.** Experimental ^{115}In WURST-QCPMG NMR spectrum of $In(NO_3)_3{\cdot}5H_2O$, the spectrum profile (with corrected background) obtained by the UWNMRSpectralShape software, and experimental ^{115}In Hahn-echo spectrum.	S10
**Figure S8.** Profiles obtained by the UWNMRSpectralShape software of the ^{115}In WURST-QCPMG NMR spectrum of $In(NO_3)_3{\cdot}5H_2O$ processed with full and cut FID	S10
**Figure S9.** Phase analysis of the measured powder diffraction pattern of $In(NO_3)_3{\cdot}5H_2O$ sample	S11
**References**	S12

Table S1. Details of NMR experimental parameters

Compound	Investigated nuclei	Spikelet separation (Hz)	WURST-sweep width (MHz)	Loops No.	sub-spectra No.	Step(s) (kHz).	D_1 (s)
$Ba(NO_3)_2$	^{137}Ba	2000	0.5	64	1	---	0.5
$BaCO_3$	^{137}Ba	5000	1	80	7	150	0.1
$BaCl_2{\cdot}2H_2O$	^{35}Cl	1000	0.5	64	1	---	4
$AlMes_3$<br>$AlMes_3{\cdot}THF$	^{27}Al	5000	1	64	5	100	2
$Pb(HCOO)_2$	^{207}Pb	2500	0.5	75	1	178	7
$Hg(CH_3COO)_2$	^{199}Hg	5000	0.75	75	1	---	1450
$Cs_2AgBiBr_6$	^{209}Bi	5000	2	12	1	---	4
$Ga(NO_3)_3{\cdot}9H_2O$<br>$Ga_{13}(NO3)_{15}(OH)_{24}$<br>$\cdot 24H_2O$	^{71}Ga	1000	1	32	1	---	2
$In(NO_3)_3{\cdot}5H_2O$	^{115}In	5000	1	16	1	---	2
$[Cl(Me)Ga(O^tBu)]_2$	^{71}Ga	5000	1	32	17	150	2

Table S2. Used external standards for NMR chemical shift referencing

Nuclei	External standard
^{137}Ba	0.5 M solution of $BaCl_2$ in D_2O (^{137}Ba: 0.0 ppm)
^{35}Cl	0.1 M solution of NaCl in D_2O (^{35}Cl: 0.0 ppm)
^{27}Al	1 M solution of $Al(NO_3)_3$ in D_2O (^{27}Al: 0.0 ppm)
^{207}Pb	solid $Pb(NO_3)_2$ (^{207}Pb: −3473.6 ppm)
^{199}Hg	The chemical shift referencing was calculated to the unified scale accordingly to IUPAC [1]
^{209}Bi	Saturated solution of $Bi(NO_3)_3{\cdot}5H_2O$ in concentrated HNO_3 (^{209}Bi: 0.0 ppm)
^{71}Ga	1 M solution of $Ga(NO_3)_3$ in D_2O (^{71}Ga: 0.0 ppm)
^{115}In	0.1 M solution of $In(NO_3)_3$ in 0.5 M of HNO_3 (^{115}In: 0.0 ppm)

Table S3. Crystallographic data of the investigated compounds

Compound	*PDF* or **CSD** code*	Space group	Reference
$Ba(NO_3)_2$	*01-070-9288*	Pa-3	[2]
$BaCO_3$	*01-074-2663*	Pnam	[3]
$BaCl_2·2H_2O$	*01-081-9342*	P21/n	[4]
$AlMes_3$	**TMEAL**	C2/c	[5]
$AlMes_3·THF$	**YEVVAZ**	P21/c	[6]
$Pb(HCOO)_2$	**BIYLAW01**	P212121	[7]
$Hg(CH_3COO)_2$	**HGACET10**	P21/a	[8]
$Cs_2AgBiBr_6$	**---**	Fm-3m	[9]
$Ga(NO_3)_3·9H_2O$	*04-016-7745*	P21/c	[10]
$Ga_{13}(NO3)_{15}(OH)_{24}·24H_2O$	*04-014-0474*	R-3	[11]
$In(NO_3)_3·5H_2O$	*04-017-7341*	C2/c	[12]

* PDF codes are denoted in italic font, CSD codes are denoted in bold font

One of the objectives specified the main text is to find positions and intensities of spikelets in a noisy spectrum so that the spectrum can then be smoothed. It should be realized that

- The noise cannot be filtered out, as its level can be higher than intensities of spikelets in some region(s) of the spectrum.
- An application of some “black box” peak-finding procedure (for example, the “findpeaks” function of Matlab®) is not practical, as it would return numerous local maxima of the noise in addition to the actual peaks.

The solution can be easily obtained, however, since the spectrum is densely and uniformly sampled. Thus, two peaks are picked to define a bin, and maxima are located in all bins of a spectrum. Subsequently the investigated spectrum is smoothed out by an interpolation. This procedure was implemented in Matlab® independently from the software described in the main text. The block diagram follows.

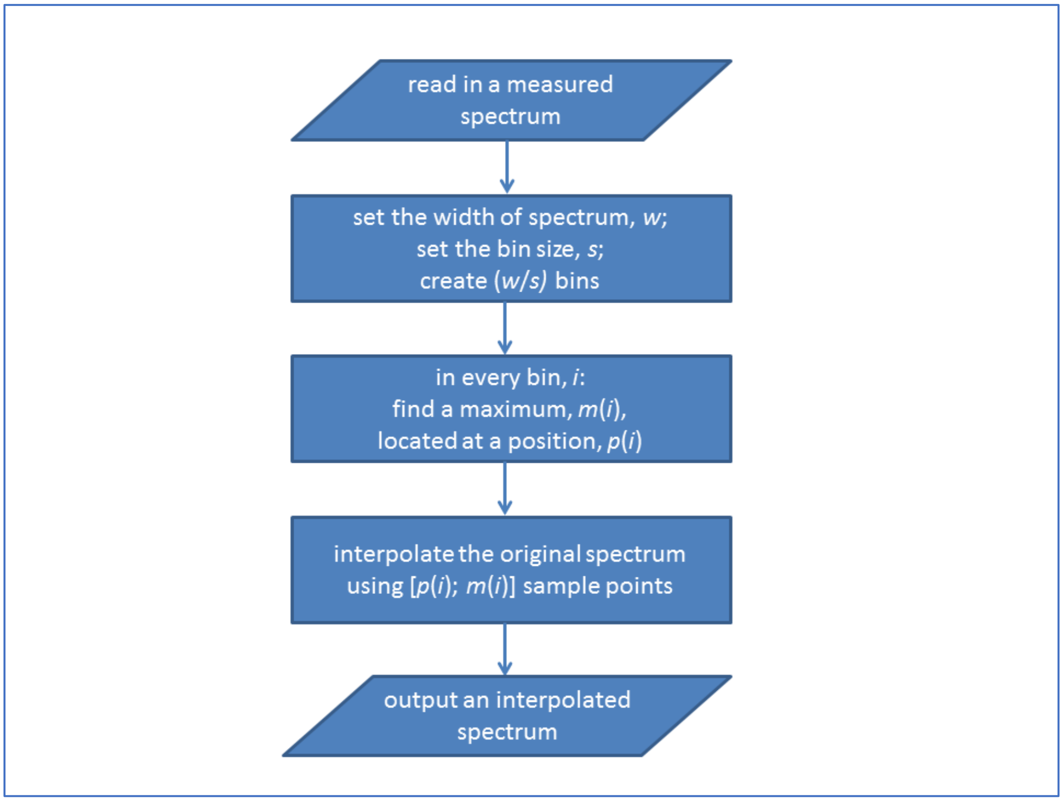

Chart S1. Description of a procedure for verifying the spectral shape.

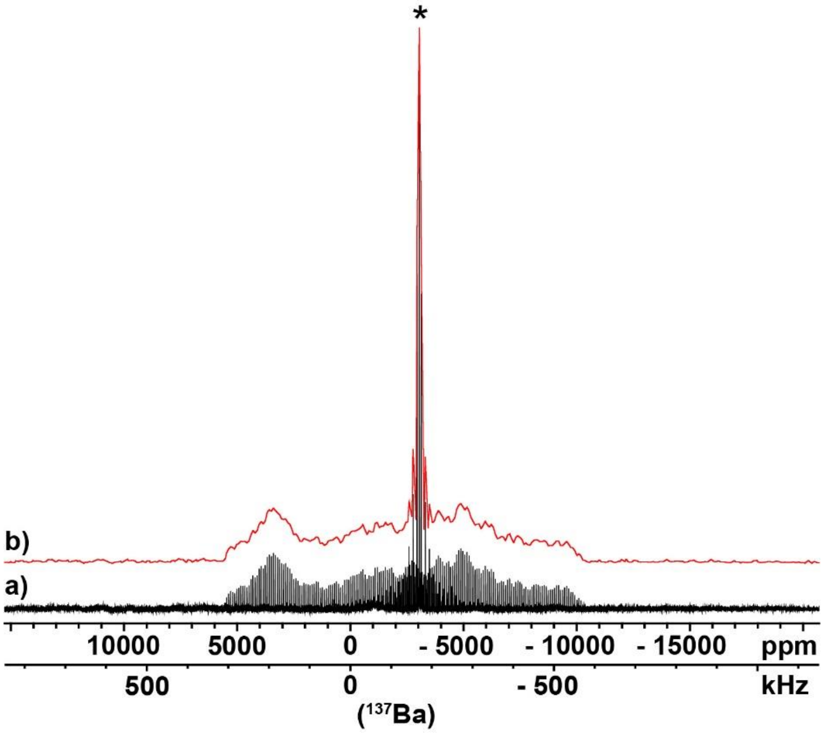

Figure S1. Full experimental ^{137}Ba WURST-QCPMG NMR spectrum of $BaCO_3$ (a) and the spectal profile (b) obtained by the UWNMRSpectralShape software. Impurity is denoted by an asterisk (*).

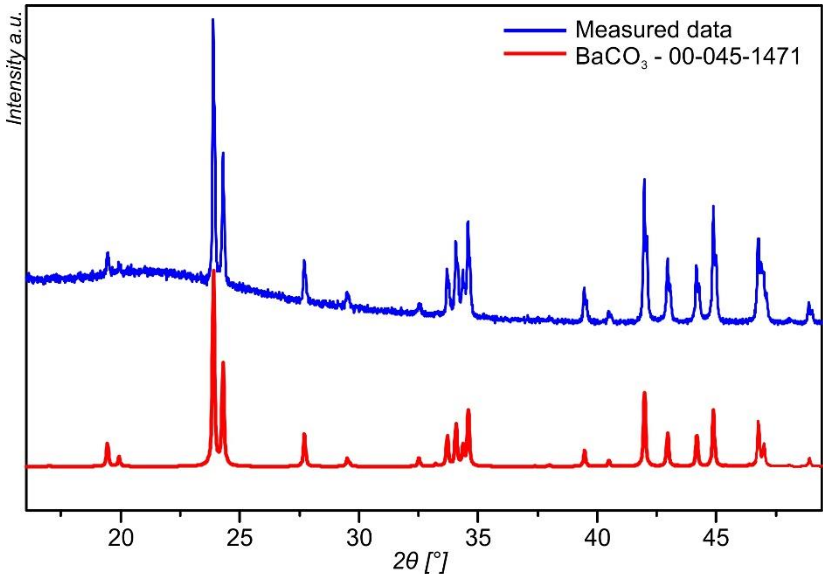

Figure S2. Phase analysis of the measured powder diffraction pattern (blue) of the $BaCO_3$ sample confirming the presence of one phase - $BaCO_3$ (calculated pattern (red), PDF card no. 00-045-1471).

.

Table S4. Comparison of NMR parameters obtained by fitting spectral envelopes provided by different approaches for chosen model compounds

Compound	Investigated nuclei	δ_{iso}, ppm	C_Q, MHz	η_Q	Ω, ppm	κ	α, deg	β, deg	γ, deg	Fitting
$Ba(NO_3)_2$	^{137}Ba	-49(5)	6.9(0.10)	0.02(0.01)	26(15)	0.80(0.20)	43(15)	11(20)	---	1
		-47(5)	6.9(0.10)	0.02(0.01)	24(15)	0.70(0.20)	53(20)	32(20)	---	2
		-49(5)	6.9(0.10)	0.02(0.01)	25(15)	0.82(0.20)	60(20)	5(20)	---	3
$BaCl_2 \cdot 2H_2O$	^{35}Cl (site A)	167(10)	2.27(0.12)	0	39(25)	-0.60(0.20)	89(20)	47(15)	49(20)	1
		164(10)	2.20(0.12)	0	43(25)	-0.60(0.20)	100(25)	30(15)	66(20)	2
		163(10)	2.28(0.12)	0	31(25)	-0.71(0.20)	93(25)	45(15)	59(20)	3
	^{35}Cl (site B)	154(10)	3.48(0.15)	0.30(0.10)	52(25)	0.20(0.20)	22(15)	12(10)	0(25)	1
		158(10)	3.49(0.15)	0.30(0.10)	47(25)	0.10(0.20)	15(15)	15(10)	5(25)	2
		149(10)	3.50(0.15)	0.29(0.10)	55(25)	0.14(0.20)	17(15)	12(10)	0(25)	3
$AlMes_3$	^{27}Al (1)	242(15)	49.2(0.50)	0.01(0.01)	126(10)	-0.99(0.20)	170(15)	0(10)	110(10)	1
		226(15)	48.8(0.50)	0.01(0.01)	118(10)	-0.99(0.20)	180(15)	0(10)	100(10)	2
		242(15)	49.3(0.50)	0.01(0.01)	134(10)	-0.99(0.20)	168(15)	0(10)	102(10)	3
$AlMes_3 \cdot THF$	^{27}Al (2)	130(10)	27.0(0.30)	0.17(0.08)	47(5)	0.05(0.08)	195(10)	83(5)	210(10)	1
		133(10)	26.8(0.30)	0.17(0.08)	51(5)	0.07(0.08)	200(10)	80(5)	215(15)	2
		133(10)	26.5(0.30)	0.16(0.08)	55(5)	0.05(0.08)	210(10)	87(5)	230(15)	3
$Hg(CH_3COO)_2$	^{199}Hg	-2485(40)	---	---	1814(65)	0.82(0.07)	---	---	---	1
		-2488(40)	---	---	1811(60)	0.82(0.07)	---	---	---	2
		-2475(40)	---	---	1800(60)	0.78(0.07)	---	---	---	3

$\delta_{iso} = (\delta_{11} + \delta_{22} + \delta_{33})/3$, $C_Q = eQV_{33}/h$, $\eta_Q = (V_{11} - V_{22})/V_{33}$, $\Omega = \delta_{11} - \delta_{33}$, $\kappa = 3(\delta_{22} - \delta_{iso})/\Omega$; Euler angles are defined in [13]. [1] NMR parameters obtained by fitting spectral envelope (created by USS software) using Bruker TopSpin software; [2] NMR parameters obtained by fitting spectral envelop (created by USS software) using Dmfit software; [3] NMR parameters obtained by fitting (using TopSpin software) spectral profile yielded by co-addition of spin echo followed by Fourier transformation.

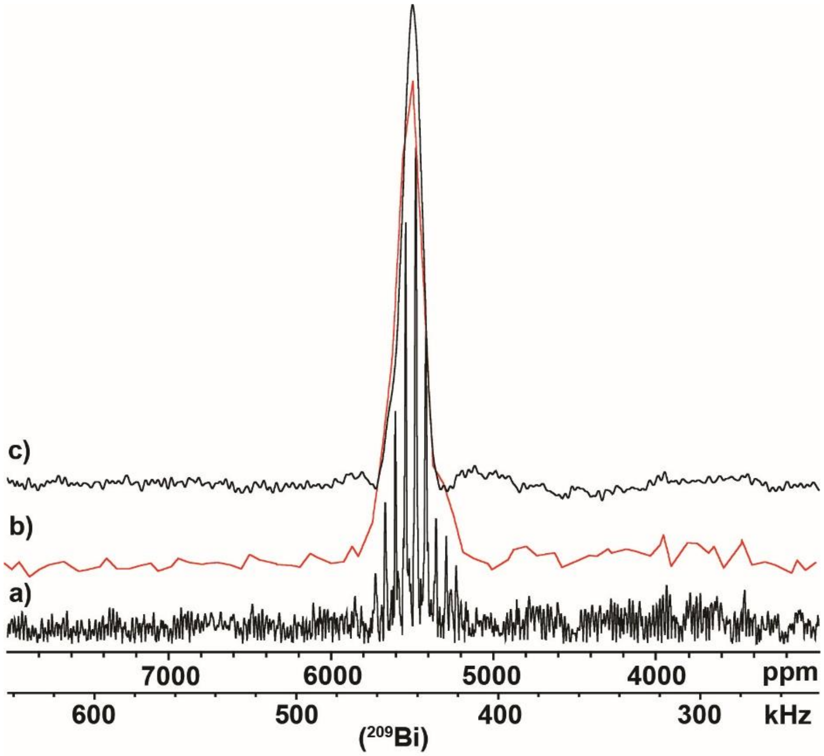

Figure S3. Experimental [209]Bi WURST-QCPMG NMR spectrum (a) of $Cs_2AgBiBr_6$, the spectrum profile (b) obtained by the UWNMRSpectralShape software, and experimental [209]Bi Hahn-echo ($^{\pi}/_{2}$-t_1-π-*aq.*, t_1 = 6.5 µs) spectrum (c).

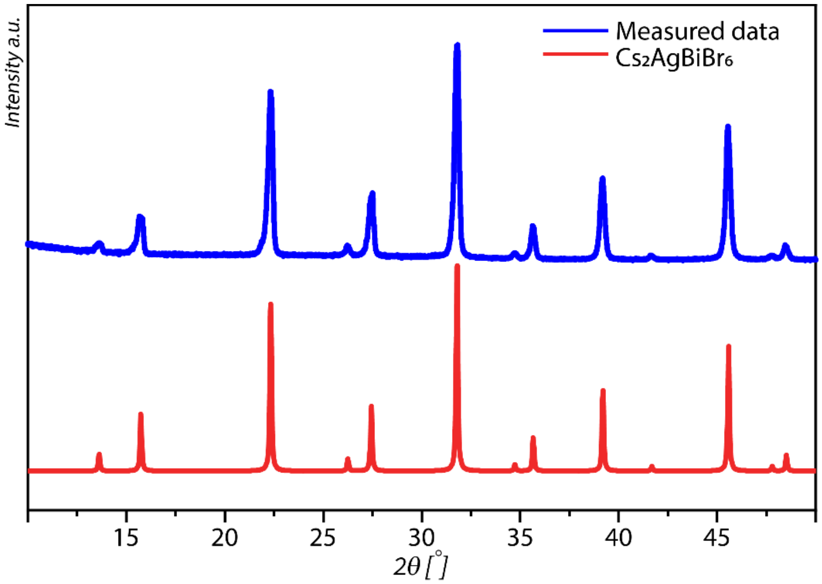

Figure S4. Phase analysis of the measured powder diffraction pattern (blue) confirming the presence of $Cs_2AgBiBr_6$ phase (calculated pattern (red))

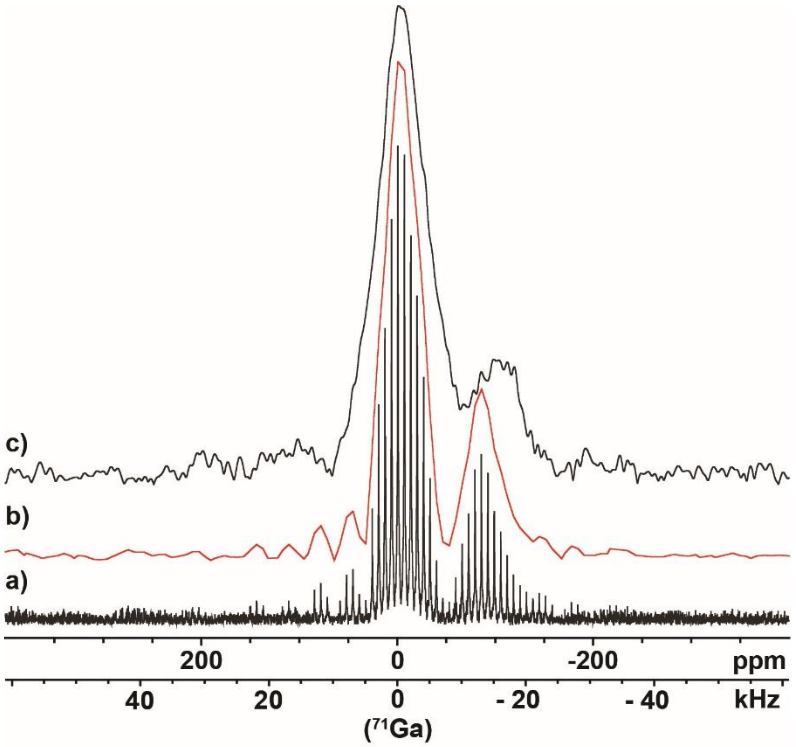

Figure S5. Experimental ^{71}Ga WURST-QCPMG NMR spectrum (a) of $Ga(NO_3)_3$, the spectrum profile (b) obtained by the UWNMRSpectralShape software, and experimental ^{71}Ga Hahn-echo ($^{\pi}/_{2}$-t_1-π-*aq.*, t_1 = 150 µs) spectrum (c).

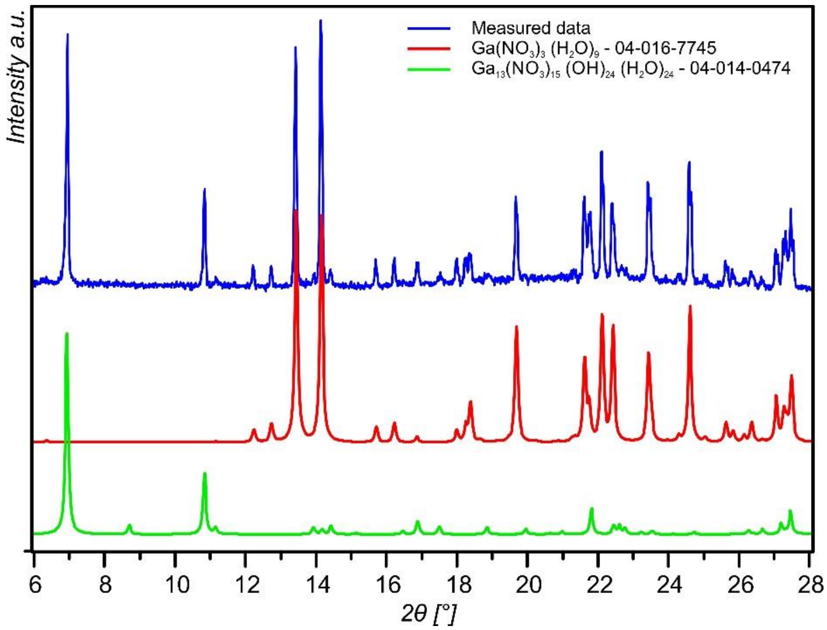

Figure S6. Phase analysis of the measured powder diffraction pattern (blue) confirming the presence of $Ga(NO_3)_3{\cdot}9H_2O$ and $Ga_{13}(NO_3)_{15}(OH)_{24}{\cdot}24H_2O$ phases (calculated patterns (red), PDF card 04-016-7745 and (green) PDF card 04-014-0474 respectively).

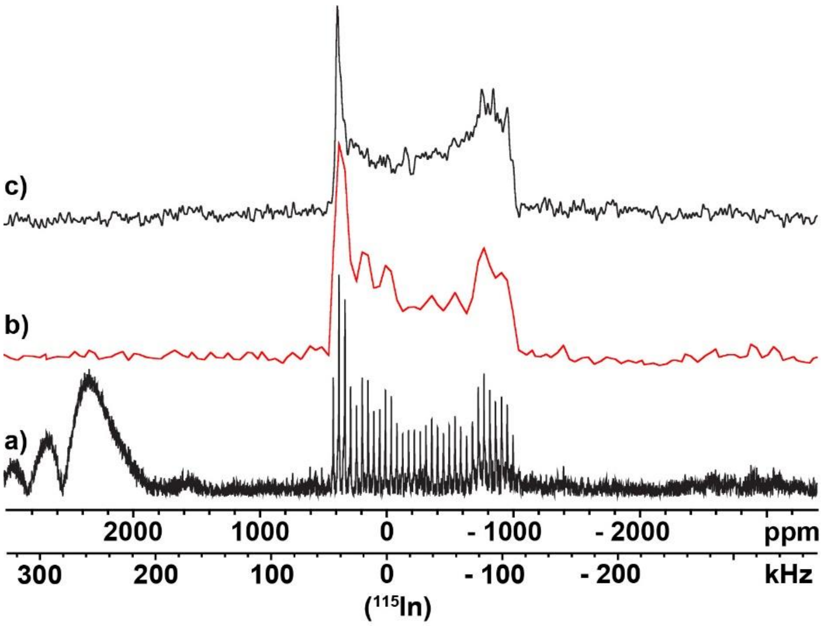

Figure S7. Experimental [115]In WURST-QCPMG NMR spectrum (a) of $In(NO_3)_3{\cdot}5H_2O$, the spectrum profile (with corrected background) (b) obtained by the UWNMRSpectralShape software, and experimental [115]In Hahn-echo ($^{\pi}/_{2}$-t_1-π-*aq.*, t_1 = 160 µs) spectrum (c).

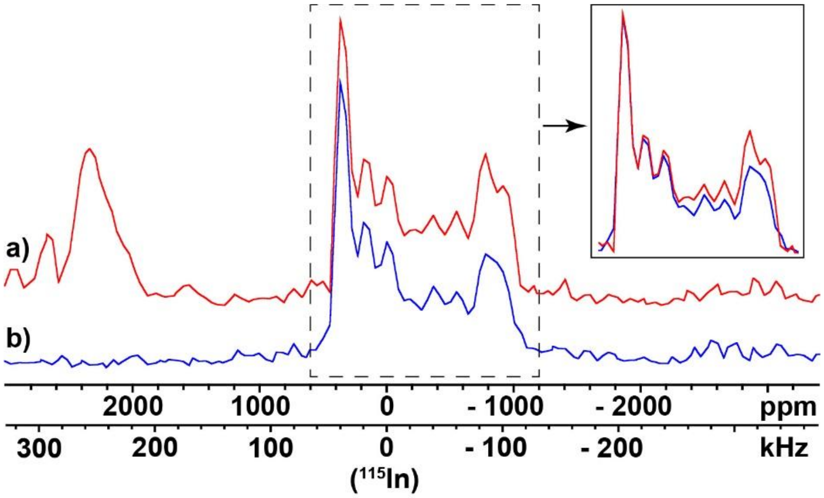

Figure S8. Profiles obtained by the UWNMRSpectralShape software of the [115]In WURST-QCPMG NMR spectrum of $In(NO_3)_3{\cdot}5H_2O$ processed with full (a) and cut (b) FID.

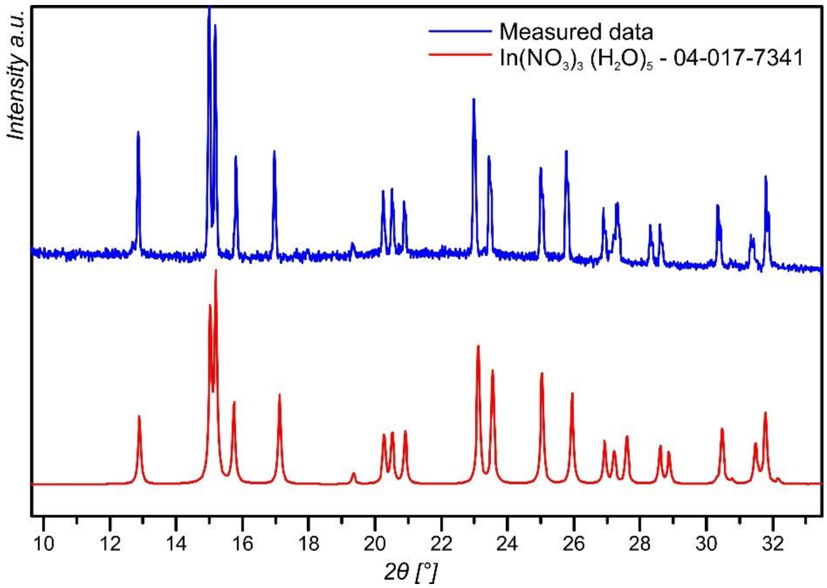

Figure S9. Phase analysis of the measured powder diffraction pattern (blue) confirming the presence of $In(NO_3)_3{\cdot}5H_2O$ phase (calculated pattern (red), PDF card no. 04-017-7341)

References

[1] R.K. Harris, E.D. Becker, S.M. Cabral de Menezes, P. Granger, R.E. Hoffman, K.W. Zilm, Further conventions for NMR shielding and chemical shifts, Pure Appl. Chem. 80 (2008) 59–84. https://doi.org/10.1351/pac200880010059.

[2] V.A. Trounov, E.A. Tserkovnaya, V.N. Gurin, M.M. Korsukova, L.I. Derkachenko, S.P. Nikanorov, Effect of centrifugation on the crystal structure of barium nitrate, Tech. Phys. Lett. 28 (2002) 351–353. https://doi.org/10.1134/1.1477015.

[3] C.M. Holl, J.R. Smyth, H.M.S. Laustsen, S.D. Jacobsen, R.T. Downs, Compression of witherite to 8 GPa and the crystal structure of BaCO3II, Phys. Chem. Miner. 27 (2000) 467–473. https://doi.org/10.1007/s002690000087.

[4] V.P. Ting, P.F. Henry, M. Schmidtmann, C.C. Wilson, M.T. Weller, Probing hydrogen positions in hydrous compounds: information from parametric neutron powder diffraction studies, Phys. Chem. Chem. Phys. 14 (2012) 6914–6921. https://doi.org/10.1039/C2CP40366H.

[5] R.G. Vranka, E.L. Amma, Crystal structure of trimethylaluminum, J. Am. Chem. Soc. 89 (1967) 3121–3126. https://doi.org/10.1021/ja00989a008.

[6] L. Brieger, A. Hermann, C. Unkelbach, C. Strohmann, Crystal structure and quantum-chemical calculations of a trimethylaluminium-THF adduct, Acta Crystallogr. Sect. E Crystallogr. Commun. 74 (2018) 267–270. https://doi.org/10.1107/S2056989018001275.

[7] A. Lanza, A. Gambirasi, M. Favaro, L. Peruzzo, M. Chiurato, F. Nestola, Re-investigation of lead(II) formate, Acta Crystallogr. Sect. C Cryst. Struct. Commun. 69 (2013) 41–43. https://doi.org/10.1107/S010827011204913X.

[8] R. Allmann, No Title, Z.Kristallogr.,Kristallgeom.,Kristallphys.,Kristallchem. 138 (1973) 366–373.

[9] A.H. Slavney, T. Hu, A.M. Lindenberg, H.I. Karunadasa, A Bismuth-Halide Double Perovskite with Long Carrier Recombination Lifetime for Photovoltaic Applications, J. Am. Chem. Soc. 138 (2016) 2138–2141. https://doi.org/10.1021/jacs.5b13294.

[10] A.D. Hendsbee, C.C. Pye, J.D. Masuda, Hexaaquagallium(III) trinitrate trihydrate, Acta Crystallogr. Sect. E. 65 (2009) i65. https://doi.org/10.1107/S1600536809028086.

[11] E. Rather, J.T. Gatlin, P.G. Nixon, T. Tsukamoto, V. Kravtsov, D.W. Johnson, A Simple Organic Reaction Mediates the Crystallization of the Inorganic Nanocluster [Ga13(μ3-OH)6(μ2-OH)18(H2O)24](NO3)15, J. Am. Chem. Soc. 127 (2005) 3242–3243. https://doi.org/10.1021/ja043520t.

[12] M.A. Malyarik, S.P. Petrosyants, A.B. Ilyukhin, Y.A. Buslaev, Polyfunctionality of the nitrate group, coordination numbers of trivalent indium in nitrate complexes, and crystal structures of $(In(NO_3)(H_2O)_5)(NO_3)_2$ and $HK_6(In(NO_3)_4(H_2O)_2)_3(NO_3)_4$, Russ. J. Inorg. Chem. (Engl. Transl.). 28 (1993) 1849–1854.

[13] W.P. Power, R.E. Wasylishen, S. Mooibroek, B.A. Pettitt, W. Danchura, Simulation of NMR powder line shapes of quadrupolar nuclei with half-integer spin at low-symmetry sites, J. Phys. Chem. 94 (1990) 591–598. https://doi.org/10.1021/j100365a019.
